# Phylogenetic Analysis of Grapevine Fanleaf Virus, Grapevine Virus A, and Grapevine Leafroll-Associated Virus 3 in Kazakhstan

**DOI:** 10.3390/microorganisms13092142

**Published:** 2025-09-12

**Authors:** Ivan G. Frolov, Karlygash P. Aubakirova, Zhibek N. Bakytzhanova, Akbota Rakhatkyzy, Laura S. Yerbolova, Nurbol N. Galiakparov

**Affiliations:** M. Aitkhozhin Institute of Molecular Biology and Biochemistry, 86 Dosmukhamedov Str., Almaty 050012, Kazakhstan; karlygashaubakirova9@gmail.com (K.P.A.); bakytzhanovazhibek@gmail.com (Z.N.B.); akbotarahatkyzy1@gmail.com (A.R.); yerbolova.laura7@gmail.com (L.S.Y.)

**Keywords:** grapevine virus, phylogenetic analysis, molecular detection, Kazakhstan, plant epidemiology

## Abstract

Grapevine viruses reduce harvests and degrade fruit quality, but their genetic diversity in Kazakhstan has remained unexplored. We collected symptomatic leaves from local vineyards and recovered eleven fragments of the coat-protein gene: one from grapevine fanleaf virus, five from grapevine virus A, and five from grapevine leafroll-associated virus 3. After Sanger sequencing, we compared these fragments with more than one thousand international counterparts to place the Kazakh strains on the global family tree. The results reveal a clear spectrum of genetic diversity that mirrors each virus’s route of spread. Grapevine virus A, which is moved both mechanically and by insects, proved the most variable; grapevine fanleaf virus, carried by dagger nematodes and pruning sap, had intermediate variability; and grapevine leafroll-associated virus 3, moved only by mealybugs and scales, was highly conserved. All Kazakh sequences fell inside established foreign lineages, showing that the viruses were imported multiple times rather than evolving locally. Grapevine virus A will require broad-coverage or multiplex PCR primers to avoid false negatives, whereas the stable leafroll virus can be monitored with a single high-sensitivity assay. Combined with vector management—mealybug control for leafroll, and nematode testing for fanleaf—these data lay the groundwork for a national clean-plant program and more resilient vineyards across Central Asia.

## 1. Introduction

Grapevine cultivation underpins a global industry valued at more than USD 300 billion per year, yet more than 80 viruses reduce yields, compromise fruit composition, and shorten vineyard life spans [1,2]. Three pathogens account for a disproportionate share of these losses: grapevine fanleaf virus (GFLV), grapevine virus A (GVA), and grapevine leafroll-associated virus 3 (GLRaV-3). Together they are estimated to cost European producers alone more than EUR 1 billion annually [3]. We use common virus names and abbreviations (GFLV, GVA, and GLRaV-3) when referring to biological agents, and we formulate taxonomic statements at the genus and family levels in accordance with current ICTV practice [4].

GFLV—species *Nepovirus foliumflabelli* (family Secoviridae) [5]—has a bipartite, positive-sense RNA genome (RNA1 ≈ 7.3 kb; RNA2 ≈ 3.8 kb) encapsidated in 30 nm icosahedral particles [6]. Its transmission is soil-borne, mediated specifically by the dagger nematode *Xiphinema index*, and secondarily by sap during grafting or pruning [7]. Characteristic “fanleaf” deformation, chlorotic mottling, and cane stunting can reduce yields by up to 80% and shorten vineyard longevity by a decade [8]. Surveys now place GFLV in every major viticultural zone of Europe, Asia, and the Americas [2,5].

GVA—species *Vitivirus alphavitis* (family Betaflexiviridae) [9]—possesses a flexuous filamentous virion (~800 nm) that encloses a single +ssRNA genome of ~7.6 kb [10,11]. It is a core component of the rugose-wood complex, inducing Kober stem-grooving and graft incompatibility that silently erode vine vigor and productivity [12]. In addition to spreading through propagation material, GVA spreads semi-persistently via several mealybugs and soft scales (e.g., *Planococcus ficus* and *Parthenolecanium corni*) [10,11]. Molecular surveys document a global distribution, with high intraspecific diversity reported from Italy [10], Iran, and neighboring post-Soviet countries [13], Australia [14] and China.

GLRaV-3—species *Ampelovirus trivitis* (family Closteroviridae) [15]—is the dominant agent of grapevine leafroll disease (GLD). Its ~18.5 kb +ssRNA genome encodes up to 13 ORFs packed in flexuous filaments that may exceed 1.8 µm [15,16]. Mealybugs and soft-scale insects transmit GLRaV-3 in a semi-persistent manner, while infected grafting material enables them to spread over long-distances [11]. Characteristic symptoms comprise downward rolling of leaves, interveinal reddening or yellowing, delayed berry ripening, and a marked yield reduction that can exceed 40% in severe infections [17]. Recent work highlights its global prevalence and genetic stability, yet transcriptomic and proteomic studies reveal profound metabolic disruptions in infected vines [18,19,20]. Historically, GFLV has been detected only sporadically in Kazakh vineyards (e.g., a single positive vine in 2009 [21]), which is consistent with our recovery of one CP fragment in this survey. Although its overall divergence is low, GLRaV-3 segregates into at least eight well-supported phylogroups (I, II, III, V, VI, VII, IX, X) [22].

Comparative analyses show that transmission mode is a primary driver of plant-virus evolutionary rate: mechanically or multi-modally transmitted viruses accumulate more substitutions and indels than their strictly vector-borne counterparts [23]. Meta-analyses of 1050 plant-RNA-virus genomes link higher Shannon entropy to broader transmission routes, with predictive models correctly classifying 85% of viruses by route solely from sequence signatures [3]. These findings suggest that GVA should display greater CP diversity than nematode-vectored GFLV, which, in turn, should exceed the highly conserved GLRaV-3.

There exists a Central Asian knowledge gap. Kazakhstan, situated along historic Silk-Road corridors, records viticulture as early as the 7th century AD [24]. Soviet-era expansion peaked near 60,000 ha; modern revival has focused on high-altitude sites in the Almaty and Turkistan provinces, with national production reaching ~340 kt in 2024 [25,26]. Despite this heritage, no sequence-based surveys of grapevine viruses have been published from Kazakhstan. The lack of local data hampers clean-plant certification, complicates quarantine decisions, and does not offer data about Central Asia’s role in global virus dissemination.

To address this gap, we generated 11 new coat protein (CP) sequences—one GFLV, five GVA, and five GLRaV-3—and analyzed them together with more than 1300 varieties from GenBank. We formulated three testable hypotheses:

**Hypothesis** **1.** *Phylogeographic integration. Kazakh CP sequences will nest within established international clades, indicating recent introductions rather than long-term local evolution.*

**Hypothesis** **2.** *Transmission-driven diversity. Genetic heterogeneity (measured as Shannon entropy, gap frequency, and other alignment metrics) will decrease in the order GVA > GFLV > GLRaV-3, reflecting the transition from frequent mechanical transmission (GVA), through mixed nematode/mechanical spread (GFLV), to strictly vector-borne dissemination (GLRaV-3).*

**Hypothesis** **3.** *Diversity versus tree resolution. Mean bootstrap support will track sequence entropy; therefore, the average support values of phylogenetic trees should decrease in the order GVA > GFLV > GLRaV-3.*

By linking transmission ecology, sequence diversity, and phylogenetic resolution, this work establishes the first molecular baseline for grapevine-virus surveillance in Central Asia and contributes comparative insight into plant-virus evolution.

## 2. Materials and Methods

### 2.1. Sample Collection and RNA Extraction

Grapevine samples were collected from various vineyards across Kazakhstan during the 2023 growing season. Excised leaf tissue was placed on ice, transported to the laboratory within 24 h, and stored at −80 °C until processing. Total RNA was extracted from grapevine leaves using a modified protocol based on a previously published method [27]. Briefly, 100 mg of grapevine leaf tissue was ground in a pre-chilled mortar with 1 mL of extraction buffer. The buffer contained 200 mM Tris-HCl (pH 8.0), 40 mM EDTA (pH 8.0), 2.8 M NaCl, 4% CTAB, and 4% PVP. We added 0.2% of fresh 2-mercaptoethanol before use. The homogenate was incubated at 65 °C for 40 min, followed by chloroform extraction. An equal volume of chloroform was added, and the mixture was centrifuged at 10,000× *g* for 10 min. The aqueous phase was transferred to a clean tube, and RNA was precipitated with ethanol. The RNA pellet was dried and resuspended in 60 µL of nuclease-free water.

### 2.2. Reverse Transcription

Multiplex RT-PCR was performed to simultaneously detect multiple grapevine viruses. Reverse transcription was carried out using virus-specific reverse primers in a two-step protocol. In the first step, a 14.5 µL reaction mixture containing 1 µL (150 ng) of total RNA, 1 µL (10 µM) of a specific reverse primer for each target, and nuclease-free water was incubated at 72 °C for 10 min and then cooled on ice for 5 min.

In the second step, the following components were added to the mixture: 4 µL of 5× RT buffer (250 mM Tris-HCl, pH 8.3, at 25 °C; 250 mM KCl; 20 mM MgCl_2_; 50 mM DTT), 0.5 µL of 10 mM dNTP mix, and 0.5 µL (100 U) of QuantumScript™ Reverse Transcriptase (MCLAB, South San Francisco, CA, USA, catalog number SSII-100). The complete reaction solution was incubated at 42 °C for 1 h. The resulting cDNA was then used as a template for PCR amplification using specific forward and reverse primers.

### 2.3. Multiplex RT-qPCR

To address the high genetic variability of grapevine viruses and ensure reliable detection, we developed a novel set of primers and hydrolysis probes [28]. The design process was based on comprehensive alignments of publicly available full-genome sequences from the NCBI GenBank database, including 100 sequences for GVA, 227 for GFLV, and 100 for GLRaV-3. Conserved regions within the coat protein gene were targeted to create degenerate primers and probes using Primux software, release of 20 July 2014 [29]. The design parameters were set to a product size of 80–300 bp, a primer melting temperature (Tm) of 56–60 °C, and a probe Tm of 67–73 °C. The in silico specificity of all oligonucleotides was subsequently verified using NCBI web-based tool Primer-BLAST [30] to prevent cross-reactivity.

Real-time PCR was conducted in a 25 µL reaction. The reaction mixture contained 2.5 µL of 10× Taq buffer, 2.5 µL of 25 mM MgCl_2_, and 0.2 mM of each dNTP. We added 0.2 µM of each forward and reverse primer for each virus and internal control (Table S1). The reaction included 0.5 U of Taq DNA polymerase and 2 µL of cDNA. Amplification was performed on a Gentier 96E PCR instrument (Tianlong Science & Technology, Xi’an, China) under the following cycling conditions: initial denaturation at 94 °C for 5 min, followed by 40 cycles at 94 °C for 30 s, 55 °C for 30 s, and 72 °C for 60 s, with a final extension at 72 °C for 10 min. Fluorescence data were collected at the end of each cycle, and data analysis was conducted using the Real-time PCR System Version 1 software (Tianlong Science & Technology, Xi’an, China).

### 2.4. Amplification and Sanger Sequencing of the Coat Protein Gene

Samples that tested positive in the RT-qPCR screen were used to amplify fragments of the coat protein (CP) gene for sequencing. The primers used for this purpose (Table S2) were designed manually to amplify either the full-length coat protein sequence (for GVA and GLRaV-3) or a partial fragment (for GFLV), based on the reference sequences listed in Table S2. Reverse transcription was performed as described in Section 2.2, but using the virus-specific reverse primers listed in Table S2. The resulting cDNA was then used as a template for PCR with Pfu DNA Polymerase (MCLAB, South San Francisco, CA, USA, cat. no. AD-205) and the corresponding forward and reverse primers (Table S2). The amplification conditions were initial denaturation at 95 °C for 3 min, followed by 35 cycles at 95 °C for 30 s, 58 °C for 30 s, and 72 °C for 1 min, and a final extension at 72 °C for 7 min.

The purified PCR products were used for Sanger sequencing. Sanger sequencing reactions were performed in a 10 µL volume containing 5–10 ng of PCR product, 3.2 µM of the appropriate forward or reverse primer (Table S2), 1 µL of BigDye™ Terminator v3.1 Cycle Sequencing Kit (Thermo Fisher Scientific, Waltham, MA, USA), and 1.5 µL of BigDye™ Sequencing Buffer. The thermal cycling conditions included initial denaturation at 96 °C for 1 min, followed by 25 cycles of 96 °C for 10 s, 50 °C for 5 s, and 60 °C for 4 min. The sequencing products were analyzed on an ABI PRISM^®^ 3500 Genetic Analyzer (Thermo Fisher Scientific, Waltham, MA, USA). This yielded one 670 bp fragment for GFLV (deposited in GenBank with accession number OR454495), five 579 bp fragments for GVA (OR454490–OR454494), and five 905 bp fragments for GLRaV-3 (OR454485–OR454489). All new sequences showed high identity to the reference isolates: ≥93% for GFLV, ≥92% for GVA, and >99% for GLRaV-3.

### 2.5. Data Analysis and Phylogenetic Methods

All computational analyses were performed in R 4.4.2 [31] with Bioconductor 3.20 [32]. Sequence alignments were generated using the MUSCLE algorithm v5 [33]. Alignment statistics (pairwise identity, gap content, Shannon entropy) were calculated with pegas 1.3 [34] and seqinr 4.2.36 [35]. The best-fit substitution model for each dataset was selected with ModelTest-NG under AICc and BIC [36]. Maximum-likelihood trees were inferred in IQ-TREE 2 with 1000 ultrafast-bootstrap replicates [37]. Trees were visualized and annotated in R using ggtree [38]. Bootstrap support values were summarized as the mean, median, and percentage of nodes with ≥50%, ≥70%, and ≥90% support. We did not assign GLRaV-3 “group” labels because the partial CP fragment analyzed here has limited power to discriminate Diaz-Lara groups, which are most reliably resolved with genome-wide data or loci such as HSP70h or the 3′UTR [22].

### 2.6. Amino-Acid Substitution Analysis

Kazakh CP sequences were translated (standard genetic code) and compared to reference proteins. Substitutions were classified as synonymous, conservative missense, non-conservative missense or stop-gain/loss based on cumulative changes in residue size, charge, polarity, and flexibility. Shannon entropy per codon position was calculated (H = −Σ p_i_ ln p_i_) and plotted in sliding windows (30 nt with a step of 15 nt; 12 nt with a step of 6 nt) to identify variability hotspots. Substitutions were mapped onto entropy profiles to locate functionally plastic versus structurally constrained regions. All alignments, phylogenetic trees, and custom R scripts are available from the corresponding author upon request.

## 3. Results

### 3.1. Alignment Metrics and Entropy Profiles

#### 3.1.1. Number of Sequences, Alignment Length, Gap, and Ambiguity Content

We assembled three coat-protein alignments comprising 466 GFLV, 367 GVA, and 478 GLRaV-3 sequences, yielding matrices of 679, 595, and 909 nt, respectively. Gaps were most frequent in GVA (2.63 ± 0.17%), intermediate in GFLV (1.54 ± 0.14%), and rarest in GLRaV-3 (0.44 ± 0.03%). Ambiguous characters were uniformly negligible (<0.15% in all cases), reflecting high overall alignment quality.

#### 3.1.2. GC-Content Ranges and Pairwise Identity Statistics

Base composition varied in concert with phylogenetic breadth: GFLV displayed a GC of 40.50–45.66% (mean 43.35%), GVA a GC of 45.71–51.93% (mean 50.33%), and GLRaV-3 a GC of 44.77–47.96% (mean 46.27%). Pairwise nucleotide identity likewise spanned a broad range in GVA (75.09–100.00%, mean 84.78%) and an intermediate range in GFLV (77.07–99.85%, mean 86.54%), and was highest in GLRaV-3 (74.78–100.00%, mean 93.94%), underscoring the gradient of sequence conservation from vector-only to mechanical transmission.

#### 3.1.3. Shannon Entropy and Indel Characteristics

**Figure 1:**
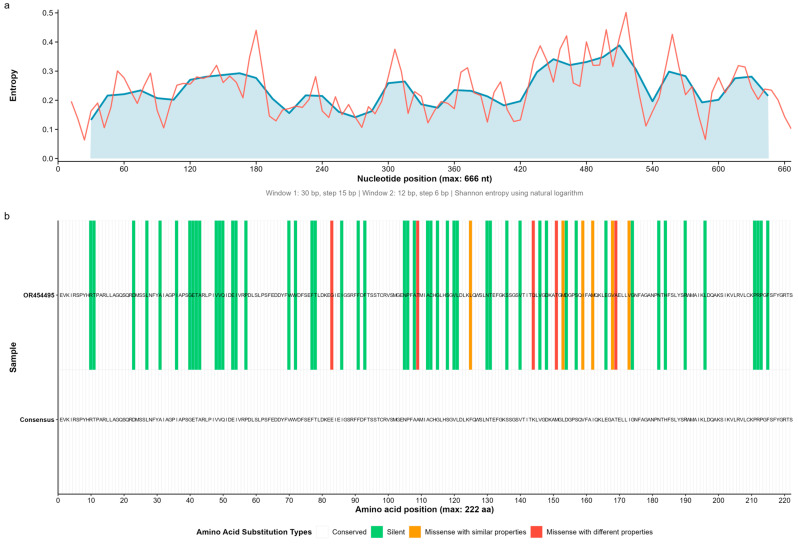
Entropy profile and amino-acid substitutions in grapevine fanleaf virus (GFLV). (**a**) Nucleotide-level Shannon entropy across a 666 nt fragment of the coat-protein gene, smoothed over two sliding windows (30 nt/step of 15 nt in blue; 12 nt/step of 6 nt in red). (**b**) Heat-map of amino-acid variation between the GFLV consensus (bottom row) and isolate OR454495 (top row). Tile colors denote the following: white—conserved (no amino-acid change); green—silent (synonymous nucleotide substitution, amino acid unchanged); orange—conservative missense (amino-acid change with similar biochemical properties); red—non-conservative missense (amino-acid change with different biochemical properties).

**Figure 2:**
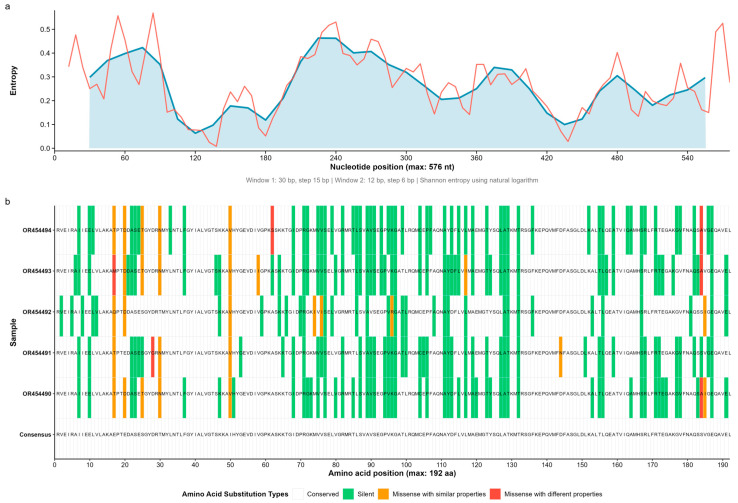
Entropy profile and amino-acid substitutions in Grapevine virus A (GVA). (**a**) Shannon entropy across a 576 nt coat-protein region, showing peaks at the 5′ and 3′ ends. Window smoothing and color coding as in Figure 1a. (**b**) Amino-acid substitution map for five GVA isolates versus the consensus. Color coding is identical to that in Figure 1b.

**Figure 3:**
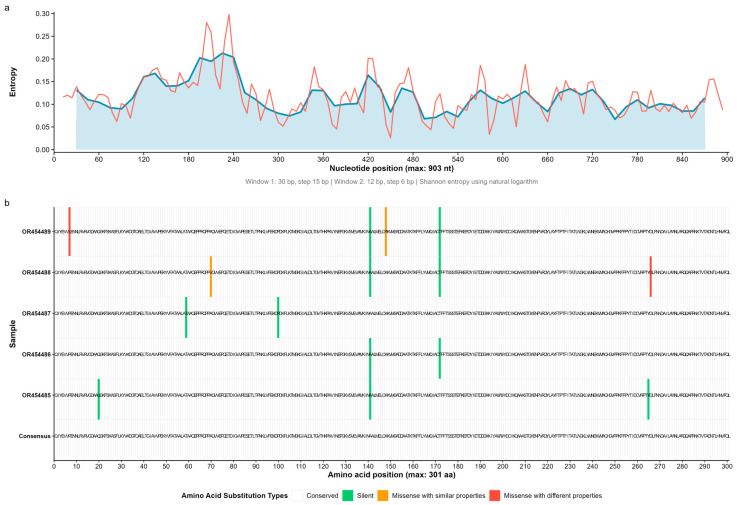
Entropy profile and amino-acid substitutions in grapevine leafroll-associated virus 3 (GLRaV-3). (**a**) Shannon entropy distribution along a 903 nt alignment of the GLRaV-3 coat-protein gene. Window smoothing and color coding as in Figure 1a. (**b**) Substitution heat-map for five Kazakh GLRaV-3 isolates against the consensus. Color coding is identical to that in Figure 1b.

Shannon entropy profiles revealed mean per-site values of 0.335 bits in GFLV (Figure 1), 0.381 bits in GVA (Figure 2), and 0.167 bits in GLRaV-3 (Figure 3), with respective maxima of 0.535, 0.623, and 0.299 bits (sliding window w = 50). Indel tracts averaged 1.56 bp (max 4) in GFLV, 2.26 bp (max 9) in GVA, and 1.00 bp (max 3) in GLRaV-3. Together, these metrics delineate a continuum of structural and compositional heterogeneity—from the moderately variable GFLV, through the highly plastic GVA, to the tightly constrained GLRaV-3.

### 3.2. Amino-Acid Substitution Patterns

#### 3.2.1. Total Substitutions per Isolate and Their Classification

Across the eleven Kazakh isolates, we observed a striking gradient in substitution load. The single GFLV isolate (OR454495) harbored 61 amino-acid changes (Figure 1); GVA isolates ranged from 41 to 63 (mean 56.6) substitutions each (Figure 2); and GLRaV-3 isolates exhibited only two–four (mean 3.0) changes (Figure 3). When partitioned by biochemical class, synonymous (“no-change”) mutations dominated (GFLV: 82.0%; GVA: 87.2% on average; GLRaV-3: 60.0% overall), conservative missense substitutions accounted for 7–19.5% of mutations, and non-conservative replacements comprised up to 8.2% (Table 1).

#### 3.2.2. Functional-Impact Categories (No, Low, and Moderate Impact)

We next scored each non-synonymous change in terms of the cumulative perturbation of size, charge, polarity, and flexibility. No high-impact (stop-codon) mutations were found. In GFLV, 6/61 (9.8%) substitutions were classified as low-impact and 5/61 (8.2%) as moderate-impact. GVA isolates averaged 11.7% low-impact and 1.8% moderate-impact changes per genome, while GLRaV-3 carried only two moderate-impact and two low-impact substitutions among 15 total replacements (13.3% functional-impact mutations).

#### 3.2.3. Summary Statistics Across All Eleven Isolates

In total, 359 amino-acid substitutions were cataloged (mean: 32.6 changes per isolate). Synonymous changes constituted 85.5% of the spectrum, low-impact missense changes 10.9%, and moderate-impact changes 3.6%. The paucity of non-conservative and moderate-impact mutations—especially in GLRaV-3—underscores pervasive purifying selection acting on the coat-protein fragment, with only GVA exhibiting sufficient mutational load to generate modest pools of potentially adaptive variation.

### 3.3. Evolutionary Model Selection and Phylogenetic Inference

#### 3.3.1. Best-Fit Substitution Models for GFLV, GVA, and GLRaV-3

Model selection using Akaike and Bayesian criteria yielded clear winners for each virus. For both GFLV and GVA, the GTR + G(4) + I model was overwhelmingly supported (AIC weights of 0.9995 and 1.0000; ΔAIC to the next-best > 19), while SYM + G(4) best described GLRaV-3 (AIC weight: 0.6940; ΔAIC: 12.0) (Tables S3–S7). BIC supported these same choices with similarly decisive weightings. Estimated gamma-shape parameters (α) were lowest for GFLV (0.67) and GVA (0.81), indicating high-rate heterogeneity, and higher for GLRaV-3 (1.15); the proportion of invariable sites was ~12% in GFLV, ~9% in GVA, and ~5% in GLRaV-3.

#### 3.3.2. ML Tree Construction and Bootstrap-Support Summaries

Maximum-likelihood trees built under these models displayed markedly different resolution. Mean bootstrap support was 84.0% for GFLV (Figure S1), 85.0% for GVA (Figure S2), and 68.3% for GLRaV-3 (Figure S3), with corresponding medians of 92.8%, 94.4%, and 64.3%. The fractions of internal nodes supported at ≥50% and ≥70% thresholds were 83.0%/65.5% (GFLV), 89.9%/74.0% (GVA), and 39.7%/14.7% (GLRaV-3). The tree lengths (substitutions per site) were 1.42 for GFLV, 1.88 for GVA, and 1.05 for GLRaV-3, and the average branch lengths mirrored this same relative pattern.

#### 3.3.3. Correlation of Model Complexity, Sequence Diversity, and Tree Resolution

To explore how substitution-model complexity, underlying sequence heterogeneity, and phylogenetic confidence interrelate, we assigned each dataset’s best-fit model a complexity score equal to the number of free parameters (including exchangeability rates, base frequencies, the gamma shape, and invariant-site proportion). The GTR + G(4) + I model selected for GVA and GFLV (12 free parameters) was markedly more complex than the SYM + G(4) model chosen for GLRaV-3 (8 parameters). We then calculated Spearman’s rank correlations among model complexity, mean per-site Shannon entropy, and mean bootstrap support across the three viruses.

We observed a near-perfect positive correlation between model complexity and entropy (ρ = 0.98, *p* = 0.02), indicating that more genetically diverse alignments require richer substitution schemes to capture their evolutionary dynamics. A similarly strong correlation emerged between sequence entropy and tree resolution (mean bootstrap support; ρ = 0.92, *p* = 0.04), confirming that higher variability yields more internally supported topologies—provided an appropriately complex model is used. Finally, model complexity itself correlated closely with bootstrap support (ρ = 0.94, *p* = 0.03), demonstrating that the statistical flexibility afforded by additional parameters translates directly into greater phylogenetic confidence.

Together, these findings underscore a unified principle: as grapevine-virus coat-protein alignments become more heterogeneous—whether through mechanical or dual transmission modes—their accurate phylogenetic reconstruction demands substitution models of sufficient complexity and yields correspondingly stronger bootstrap support.

#### 3.3.4. Possible Geographic Origins of the Kazakh Isolates

The single Kazakh GFLV isolate (OR454495) falls robustly within a North American clade, clustering with high bootstrap support alongside three U.S. and one Canadian sequence (Figure S1). The tight genetic affinity and short branch lengths linking these isolates suggest a recent introduction of GFLV into Kazakhstan via imported planting material or nursery stock originating from North America, rather than long-standing endemic evolution in Central Asia.

In contrast, the five Kazakh GVA isolates partition into two phylogenetically and geographically distinct lineages (Figure S2). Four isolates (OR454490, OR454491, OR454493, and OR454494) are embedded within a widely dispersed “cosmopolitan” clade containing sequences from Azerbaijan, Turkey, Greece, Ukraine, and the United States, consistent with multiple, broad-scale movements through international nursery exchanges. The fifth isolate (OR454492) groups separately with sequences from Australia, Russia, Armenia and China, indicating an independent introduction event—potentially via eastern Eurasian or Pacific-rim import pathways.

By contrast, all five Kazakh GLRaV-3 isolates occupy poorly supported, polytomous regions of the global tree, lacking stable association with any well-defined geographic cluster (Figure S3). This diffuse placement reflects the extreme sequence conservation and clonal propagation of GLRaV-3 in the coat-protein fragment, which limits resolution of origin. Future whole-genome analyses or the inclusion of more variable genomic regions will be required to pinpoint the routes by which GLRaV-3 has entered and circulated within Kazakhstan.

## 4. Discussion

### 4.1. Molecular–Epidemiological Insight

This work provides the first molecular-epidemiological insight into GFLV, GVA and GLRaV-3 in Kazakhstan and, by extension, Central Asia. All eleven coat-protein sequences intermingled with previously described international lineages, demonstrating that these viruses have been introduced repeatedly rather than diversified locally. Three principal patterns emerge:

1. Transmission mode governs genetic diversity. Sequence variability—quantified by Shannon entropy, gap frequency and amino-acid substitution load—was highest in GVA, intermediate in GFLV, and lowest in GLRaV-3. This gradient mirrors the shift from frequent mechanical and mixed-vector transmission (GVA), through nematode-plus-mechanical spread (GFLV), to strictly vector-borne dissemination (GLRaV-3). The data therefore support the view that transmission ecology, rather than genome size, is the principal driver of evolutionary rate in plant RNA viruses [23,39].
2. Phylogenetic structure reflects recent trade pathways. The single Kazakh GFLV isolate nests in a well-supported North American clade, implicating modern nursery imports rather than ancient Silk-Road movement. Five GVA isolates are grouped into two well-supported but geographically distinct groups, indicating at least two independent introduction events via disparate supply chains. Low CP variability in GLRaV-3 precludes fine-scale origin tracing, consistent with its globally clonal population structure. We acknowledge that GLRaV-3 diversity is organized into well-defined phylogenetic groups (I–IX and supergroups), even though our CP-based analysis cannot reliably assign our isolates to specific groups [22].
3. Functional constraints are pervasive. Across 359 amino-acid substitutions, 85% were synonymous and only 3.6% reached a moderate functional impact; no high-impact (stop-gain) variants were detected. This underscores strong purifying selection on the coat protein, particularly in GLRaV-3, where only four missense changes were observed.

### 4.2. Practical Implications

Calibrate diagnostics to diversity: GLRaV-3 conservation permits highly sensitive singleplex RT-qPCR assays, whereas the mutational plasticity of GVA necessitates degenerate or multiplex primer sets.

Reinforce vector and import management: We should prioritize mealybug control for GLRaV-3; implement nematode testing and sanitation of propagation tools for GFLV and GVA, respectively; and enhance phytosanitary scrutiny of North American planting material to curb GFLV ingress.

Focus surveillance on informative sites: We should treat high-entropy regions in GVA and moderate-impact substitutions in GFLV as logical markers for monitoring emergent variants with altered virulence or transmission efficiency.

### 4.3. Limitations and Future Work

Our analysis is restricted to partial CP fragments and a small sample set; whole-genome sequencing, broader geographic coverage, and contemporaneous vector surveys are needed to quantify recombination, date introduction events, and link viral genotypes to specific insect or nematode populations. Functional assays should test whether the moderate-impact substitutions identified here influence fitness in plants or transmission efficiency. In addition, once larger time-structured datasets are available, phylodynamic approaches (e.g., discrete-trait phylogeography and continuous spatiotemporal diffusion) [40] will be used to infer rates and routes of spread and to estimate the most probable ancestral areas for Kazakh lineages.

## 5. Conclusions

This study supplies the first molecular evidence of GFLV, GVA, and GLRaV-3 in Kazakhstan and tests three a priori hypotheses on their evolution.

1. Hypothesis 1: Phylogeographic integration. All Kazakh coat-protein sequences were nested within established international clades, confirming recent introductions rather than endemic diversification.
2. Hypothesis 2: Transmission-driven diversity. Genetic variability declined in the expected order, GVA > GFLV > GLRaV-3, mirroring the gradient from frequent mechanical spread to strictly vector-borne transmission.
3. Hypothesis 3: Diversity versus tree resolution. Mean bootstrap support tracked sequence entropy—high in GVA (85%) and GFLV (84%), and low in GLRaV-3 (68%)—validating the predicted link between variability and phylogenetic resolution.

Practically, these data support the need for (i) degenerate or multiplex primers for highly variable GVA, (ii) singleplex high-sensitivity assays for conserved GLRaV-3, and (iii) strengthened quarantines for planting material—particularly from North America—for GFLV. These insights establish a surveillance baseline for Central Asia and illustrate how transmission ecology shapes plant-virus evolution.

## Figures and Tables

**Table 1:** Summary of amino-acid substitution types in Kazakh grapevine virus isolates.

Virus	Accession ID	Total Substitutions	Synonymous (%)	Conservative (%)	Non-Conservative (%)
GFLV	OR454495	61	50 (82.0)	6 (9.8)	5 (8.2)
GVA	OR454490	61	54 (88.5)	6 (9.8)	1 (1.6)
	OR454491	56	51 (91.1)	4 (7.1)	1 (1.8)
	OR454492	41	33 (80.5)	8 (19.5)	0 (0.0)
	OR454493	63	55 (87.3)	6 (9.5)	2 (3.2)
	OR454494	62	55 (88.7)	5 (8.1)	2 (3.2)
GLRaV-3	OR454485	3	3 (100.0)	0 (0.0)	0 (0.0)
	OR454486	2	2 (100.0)	0 (0.0)	0 (0.0)
	OR454487	2	2 (100.0)	0 (0.0)	0 (0.0)
	OR454488	4	2 (50.0)	1 (25.0)	1 (25.0)
	OR454489	4	2 (50.0)	1 (25.0)	1 (25.0)

## Data Availability

All sequence data analyzed in this study are publicly available. The reference coat-protein sequences for GFLV, GVA, and GLRaV-3 were downloaded from NCBI GenBank (release 266.0; search date: 10 June 2025). The eleven new Kazakh sequences obtained in this study have been deposited in GenBank under accession numbers OR454485–OR454495, as listed in the manuscript.

## Supplementary Materials

The following supporting information can be downloaded at: https://www.mdpi.com/article/10.3390/microorganisms13092142/s1, Figure S1: Maximum-likelihood (ML) phylogenetic tree and p-distance heatmap for Grapevine fanleaf virus (GFLV) isolates, including the single Kazakh sequence (OR454495) and 466 international references; Figure S2: Maximum-likelihood (ML) phylogenetic tree and p-distance heatmap for Grapevine virus A (GVA) isolates, including five Kazakh sequences (OR454490–OR454494) and 367 international references; Figure S3: Maximum-likelihood (ML) phylogenetic tree and p-distance heatmap for Grapevine leafroll-associated virus 3 (GLRaV-3) isolates, including five Kazakh sequences (OR454485–OR454489) and 478 international references; Table S1: Oligonucleotides and probes used in multiplex real-time RT-qPCR screening; Table S2: Detailed model-selection results for GFLV coat-protein sequences; Table S3: Detailed model-selection results for GVA coat-protein sequences; Table S4: Detailed model-selection results for GLRaV-3 coat-protein sequences; Table S5: Summary of best-fit nucleotide-substitution models across the three viruses; Table S6: Comparative alignment statistics and ultrafast-bootstrap metrics for GFLV, GVA, and GLRaV-3. Table S7: Comparative alignment and bootstrap statistics for three grapevine viruses.

## Abbreviations

The following abbreviations are used in this manuscript:

AIC	Akaike Information Criterion
AICc	Corrected Akaike Information Criterion
AICw	AIC weight
BIC	Bayesian Information Criterion
BLAST	Basic Local Alignment Search Tool
bp	Base pair
BS	Bootstrap support
CP	Coat protein
CTAB	Cetyl-trimethyl-ammonium bromide
df	Degrees of freedom
G(4)	Gamma-distributed rate heterogeneity with four categories
GFLV	Grapevine fanleaf virus
GLRaV-3	Grapevine leafroll-associated virus 3
GVA	Grapevine virus A
H	Shannon entropy (bits)
IC	Internal control (18S rRNA)
kb	Kilobase (10^3^ bp)
kt	Kiloton (10^3^ t, grape production)
logLik	Log-likelihood
ML	Maximum likelihood
nt	Nucleotide
OIV	International Organisation of Vine and Wine
ORF	Open reading frame
PVP	Polyvinylpyrrolidone
RNA	Ribonucleic acid
RT-PCR	Reverse-transcription polymerase chain reaction
RT-qPCR	Quantitative RT-PCR

## References

[1] Fust C., Lameront P., Shabanian M., Song Y., Abou Kubaa R., Bester R., Maree H.J., Al Rwahnih M., Meng B. (2025). Grapevine leafroll-associated virus 3: A global threat to the grapevine and wine industries but a gold mine for scientific discovery. J. Exp. Bot..

[2] Kubina J., Hily J.-M., Mustin P., Komar V., Garcia S., Martin I.R., Poulicard N., Velt A., Bonnet V., Mercier L. (2022). Characterization of Grapevine fanleaf virus isolates in ‘Chardonnay’ vines exhibiting severe and mild symptoms in two vineyards. Viruses.

[3] Wardeh M., Pilgrim J., Hui M., Kotsiri A., Baylis M., Blagrove M.S.C. (2024). Features that matter: Evolutionary signatures can predict viral transmission routes. PLoS Pathog..

[4] Rubino L., Abrahamian P., An W., Aranda M.A., Ascencio-Ibañez J.T., Bejerman N., Blouin A.G., Candresse T., Canto T., Cao M. (2025). Summary of taxonomy changes ratified by the International Committee on Taxonomy of Viruses from the Plant Viruses Subcommittee, 2025. J. Gen. Virol..

[5] International Committee on Taxonomy of Viruses (ICTV) ICTV Report Chapter: Family *Secoviridae*, Genus *Nepovirus*. https://ictv.global/report/chapter/secoviridae/secoviridae/nepovirus.

[6] Choi J., Pakbaz S., Yepes L.M., Cieniewicz E.J., Schmitt-Keichinger C., Labarile R., Minutillo S.A., Heck M., Hua J., Fuchs M. (2023). Grapevine fanleaf virus RNA1-encoded proteins 1A and 1BHel suppress RNA silencing. Mol. Plant–Microbe Interact..

[7] CABI (2022). Xiphinema index (fan-leaf virus nematode). Invasive Species Compendium.

[8] M’Rabet Samaali B., Mougou Hamdane A., Toumi A., Dhaouadi S., Kallel S. (2022). Transmission and translocation of Grapevine fanleaf virus (GFLV) from *Vitis vinifera* L. pollen to seeds and grape. Arch. Phytopathol. Plant Prot..

[9] International Committee on Taxonomy of Viruses (ICTV) ICTV Report Chapter: Family Betaflexiviridae (Interim), Genus *Vitivirus*. https://ictv.global/report/chapter/betaflexiviridae/betaflexiviridae.

[10] Caruso A.G., Bertacca S., Ragona A., Matić S., Davino S., Panno S. (2022). Evolutionary analysis of Grapevine virus A: Insights into its dispersion in Sicily (Italy). Agriculture.

[11] Hommay G., Beuve M., Herrbach É. (2022). Transmission of grapevine leafroll-associated viruses and Grapevine virus A by vineyard-sampled soft scales (*Parthenolecanium corni*, Hemiptera: Coccidae). Viruses.

[12] Vončina D., Jagunić M., De Stradis A., Diaz-Lara A., Al Rwahnih M., Šćepanović M., Almeida R.P.P. (2024). New host plant species of Grapevine virus A identified with vector-mediated infections. Plant Dis..

[13] Gharouni Kardani S., Karimi Shahri M.R., Pakbaz S. (2023). Phylogenetic analysis of Grapevine virus A from vineyards of Khorasan Razavi Province (Iran) based on partial ORF5 gene. Appl. Res. Plant Prot..

[14] Wu Q., Kinoti W.M., Habili N., Tyerman S.D., Rinaldo A., Constable F.E. (2024). Genetic diversity of Grapevine virus A in three Australian vineyards using amplicon high-throughput sequencing (Amplicon-HTS). Viruses.

[15] International Committee on Taxonomy of Viruses (ICTV) ICTV Report Chapter: Family *Closteroviridae*, Genus *Ampelovirus*. https://ictv.global/report/chapter/closteroviridae/closteroviridae/ampelovirus.

[16] Gazel M., Tunç B., Elçi E., Çağlayan K. (2022). Incidence and genetic diversity of grapevine leafroll-associated virus 3 (GLRaV-3) isolates in Turkey. Physiol. Mol. Plant Pathol..

[17] Cabaleiro C., Pesqueira A.M., García-Berrios J.J. (2023). Assessment of symptoms of grapevine leafroll disease and relationship with yield and quality of Pinot Noir grape must in a 10-year study period. Plants.

[18] Mostert I., Bester R., Burger J.T., Maree H.J. (2023). Investigating protein–protein interactions between grapevine leafroll-associated virus 3 and *Vitis vinifera*. Phytopathology.

[19] Hančević K., Čarija M., Radić Brkanac S., Gaši E., Likar M., Zdunić G., Regvar M., Radić T. (2023). Grapevine leafroll-associated virus 3 in single and mixed infections triggers changes in the oxidative balance of four grapevine varieties. Int. J. Mol. Sci..

[20] Song Y., Hanner R.H., Meng B. (2022). Transcriptomic analyses of grapevine leafroll-associated virus 3 infection in leaves and berries of ‘Cabernet Franc’. Viruses.

[21] Ryabushkina N., Askapuly A., Stanbekova G., Galiakparov N., Feldmann F., Alford D.V., Furk C. (2009). First report of three grapevine viruses in Kazakhstan. Crop Plant Resistance to Biotic and Abiotic Factors: Current Potential and Future Demands; Proceedings of the 3rd International Symposium on Plant Protection and Plant Health in Europe (ISPPHE), Julius Kühn-Institut, Berlin-Dahlem, Germany, 14–16 May 2009.

[22] Diaz-Lara A., Klaassen V., Stevens K., Sudarshana M.R., Rowhani A., Maree H.J., Chooi K.M., Blouin A.G., Habili N., Song Y. (2018). Characterization of Grapevine Leafroll-Associated Virus 3 Genetic Variants and Application towards RT-qPCR Assay Design. PLoS ONE.

[23] Butković A., González R. (2022). A brief view of factors that affect plant virus evolution. Front. Virol..

[24] McGovern P.E. (2019). Ancient Wine: The Search for the Origins of Viniculture.

[25] International Organisation of Vine and Wine (OIV) (2025). State of the World Vine & Wine Sector in 2024.

[26] Shepard W. How Kazakhstan is Becoming the Next Frontier for World-Class Wine. Forbes. 29 February 2016. https://www.forbes.com/sites/wadeshepard/2016/02/29/could-kazakhstan-become-the-next-frontier-for-world-class-wine/.

[27] Aubakirova K.P., Bakytzhanova Z.N., Erbolova L.S., Rakhhatkyzy A., Galiakparov N.N. (2024). An improved method of RNA isolation for molecular diagnostics of fruit and berry viruses. Izdenister Natizheler–Issled. Rezult..

[28] Galiakparov N.N., Aubakirova K.P., Yerbolova L.S., Rakhatkyzy A., Bakytzhanova Z.N. (2025). Set of Synthetic Oligonucleotides and Probes for Identification and Detection of Grapevine Viruses by Real-Time PCR. Kazakhstan Patent.

[29] Hysom D.A., Naraghi-Arani P., Elsheikh M., Carrillo A.C., Williams P.L., Gardner S.N. (2012). Skip the Alignment: Degenerate, Multiplex Primer and Probe Design Using K-mer Matching Instead of Alignments. PLoS ONE.

[30] Ye J., Coulouris G., Zaretskaya I., Cutcutache I., Rozen S., Madden T. (2012). Primer-BLAST: A tool to design target-specific primers for polymerase chain reaction. BMC Bioinform..

[31] R Core Team (2024). R: A Language and Environment for Statistical Computing.

[32] Huber W., Carey V.J., Gentleman R., Anders S., Carlson M., Carvalho B.S., Corrada Bravo H., Davis S., Gatto L., Girke T. (2015). Orchestrating high-throughput genomic analysis with Bioconductor. Nat. Methods.

[33] Edgar R.C. (2004). MUSCLE: Multiple sequence alignment with high accuracy and high throughput. Nucleic Acids Res..

[34] Paradis E. (2010). pegas: An R package for population genetics with an integrated–modular approach. Bioinformatics.

[35] Charif D., Lobry J.R., Bastolla U., Porto M., Roman E., Vendruscolo M. (2007). SeqinR 1.0-2: A contributed package to the R project for statistical computing devoted to biological sequences retrieval and analysis. Structural Approaches to Sequence Evolution.

[36] Darriba D., Posada D., Kozlov A.M., Stamatakis A., Morel B., Flouri T. (2020). ModelTest-NG: A new and scalable tool for the selection of DNA and protein evolutionary models. Mol. Biol. Evol..

[37] Minh B.Q., Schmidt H.A., Chernomor O., Schrempf D., Woodhams M.D., Von Haeseler A., Lanfear R. (2020). IQ-TREE 2: New models and efficient methods for phylogenetic inference in the genomic era. Mol. Biol. Evol..

[38] Yu G., Smith D.K., Zhu H., Guan Y., Lam T.T. (2017). ggtree: An R package for visualization and annotation of phylogenetic trees. Methods Ecol. Evol..

[39] Elena S.F., Fraile A., García-Arenal F. (2014). Evolution and Emergence of Plant Viruses. Adv. Virus Res..

[40] Cabrera Mederos D., Giolitti F., Torres C., Portal O. (2019). Distribution and phylodynamics of papaya ringspot virus on *Carica papaya* in Cuba. Plant Pathol..

