# Supplementary material for: Phylogenetic Analysis of Grapevine Fanleaf Virus, Grapevine Virus A, and Grapevine Leafroll-Associated Virus 3 in Kazakhstan
Source: Microorganisms. 2025 Sep 12;13(9):2142. doi: 10.3390/microorganisms13092142 (PMC12472556; doi:10.3390/microorganisms13092142)
Supplement: Supplementary file 1 [file microorganisms-13-02142-s001.zip › microorganisms-3808284-supplementary.pdf]

**Table S1.** Oligonucleotides and probes used in the multiplex real-time RT-qPCR screen.

| Virus / control | Primer / probe | Sequence (5′ → 3′) | Fluoro-phore | Quencher |
|---|---|---|---|---|
| GVA | Forward | ATG GCW CAY TAC GCC AAG A | – | – |
| | Reverse | CCG ACC AAG GCG ATG TAC | – | – |
| | Probe | AGA GCG GCT ACG ACC GAA MTA TGT ACC T | FAM | BHQ-1 |
| GLRaV-3 | Forward | CTG CTG CTT CGA CGG GYT | – | – |
| | Reverse | GTG CCA TAA CCT TYT CRT TCAT | – | – |
| | Probe | ACC CGG TAA GGC AGT ATC TAG CGT ACT TCA | JOE | BHQ-2 |
| GFLV | Forward | GGG CCA YT GTG GAA AGR CTY | – | – |
| | Reverse | YRT CAT ACC ACT TCC TCC ARG T | – | – |
| | Probe | AGT CGA GAG CTG CGG CAC TCT TTG CCG | ROX | BHQ-2 |
| 18S rRNA (IC*) | Forward | GAG CCT GAG AAA CGG CTA CC | – | – |
| | Reverse | TGT CAC TAC CTC CCC GTG T | – | – |
| | Probe | TCC AAG GAA GGC AGC AGG CG CGC AA | Cy5.5 | BHQ-3 |

* – Internal Control.

**Table S2.** Primers used for amplification and sanger sequencing of the coat protein gene.

| Primer | Sequence (5′ → 3′) | Reference sequences | Position on reference |
|---|---|---|---|
| GVA_CP_F | GAAGACATATGGCACACTACGCAAG | NC_003604 | 6406-6430 |
| GVA_CP_R | AAACGTGGATCCACCCGCGAGAAACG | | 70497024 |
| GFLV_CP_F | GTTAGTGAGTGGAACGGGAC | NC_003623 | 2801-2820 |
| GFLV_CP_R | TTTTAACTCGAGATACCCTAGACTG | | 3579-3555 |
| GLARV3_CP_F | GAACCATATGGCATTTGAACTGAAATTAG | NC_004667 | 14262-13290 |
| GFLV3_CP_R | TAGACCTCGAGCGTAGCTACTTCTTTTGC | | 14226-14198 |

**Table S3.** Detailed model selection results for *GFLV* sequences.

| Model | df | logLik | AIC | AICw | BIC | ΔAIC | ΔBIC |
|---|---|---|---|---|---|---|---|
| JC | 929 | -48110.67 | 98079.35 | 0 | 102279.01 | 19823.77 | 19778.57 |
| JC+I | 930 | -46732.09 | 95324.18 | 0 | 99528.36 | 17068.61 | 17027.92 |
| JC+G(4) | 930 | -42607.72 | 87075.44 | 0 | 91279.62 | 8819.86 | 8779.18 |
| JC+G(4)+I | 931 | -42590.23 | 87042.45 | 0 | 91251.15 | 8786.88 | 8750.71 |
| F81 | 932 | -48245.62 | 98355.25 | 0 | 102568.47 | 20099.67 | 20068.03 |
| F81+I | 933 | -46857.48 | 95580.95 | 0 | 99798.69 | 17325.38 | 17298.25 |
| F81+G(4) | 933 | -42687.84 | 87241.68 | 0 | 91459.42 | 8986.11 | 8958.98 |
| F81+G(4)+I | 934 | -42671.92 | 87211.83 | 0 | 91434.09 | 8956.26 | 8933.65 |
| K80 | 930 | -44164.52 | 90189.05 | 0 | 94393.23 | 11933.47 | 11892.79 |
| K80+I | 931 | -42761.57 | 87385.15 | 0 | 91593.85 | 9129.57 | 9093.41 |
| K80+G(4) | 931 | -38465.60 | 78793.20 | 0 | 83001.90 | 537.62 | 501.46 |
| K80+G(4)+I | 932 | -38442.25 | 78748.50 | 0 | 82961.72 | 492.93 | 461.28 |
| HKY | 933 | -44206.48 | 90278.96 | 0 | 94496.70 | 12023.39 | 11996.26 |
| HKY+I | 934 | -42781.07 | 87430.14 | 0 | 91652.40 | 9174.56 | 9151.96 |
| HKY+G(4) | 934 | -38353.00 | 78574.00 | 0 | 82796.26 | 318.42 | 295.82 |
| HKY+G(4)+I | 935 | -38317.09 | 78504.18 | 0 | 82730.96 | 248.60 | 230.52 |
| SYM | 934 | -43821.02 | 89510.04 | 0 | 93732.30 | 11254.47 | 11231.86 |

| | | | | | | | |
|---|---|---|---|---|---|---|---|
| SYM+I | 935 | -42455.44 | 86780.87 | 0 | 91007.66 | 8525.30 | 8507.22 |
| SYM+G(4) | 935 | -38305.85 | 78481.71 | 0 | 82708.49 | 226.13 | 208.05 |
| SYM+G(4)+I | 936 | -38269.91 | 78411.82 | 0 | 82643.12 | 156.24 | 142.68 |
| GTR | 937 | -43881.41 | 89636.81 | 0 | 93872.63 | 11381.24 | 11372.20 |
| GTR+I | 938 | -42496.67 | 86869.35 | 0 | 91109.69 | 8613.77 | 8609.25 |
| GTR+G(4) | 938 | -38223.15 | 78322.30 | 0 | 82562.64 | 66.73 | 62.21 |
| GTR+G(4)+I | 939 | -38188.79 | 78255.58 | 1.0000 | 82500.44 | 0 | 0 |

**Table S4.** Detailed model selection results for *GVA* sequences.

| Model | df | logLik | AIC | AICw | BIC | ΔAIC | ΔBIC |
|---|---|---|---|---|---|---|---|
| JC | 731 | -34214.18 | 69890.35 | 0 | 73098.39 | 13871.73 | 13827.84 |
| JC+I | 732 | -32379.83 | 66223.66 | 0 | 69436.08 | 10205.03 | 10165.54 |
| JC+G(4) | 732 | -29212.10 | 59888.20 | 0 | 63100.63 | 3869.58 | 3830.08 |
| JC+G(4)+I | 733 | -29165.55 | 59797.11 | 0 | 63013.92 | 3778.48 | 3743.38 |
| F81 | 734 | -34186.51 | 69841.01 | 0 | 73062.21 | 13822.39 | 13791.67 |
| F81+I | 735 | -32335.90 | 66141.80 | 0 | 69367.39 | 10123.18 | 10096.85 |
| F81+G(4) | 735 | -29190.71 | 59851.42 | 0 | 63077.01 | 3832.80 | 3806.47 |
| F81+G(4)+I | 736 | -29138.63 | 59749.27 | 0 | 62979.25 | 3730.65 | 3708.70 |
| K80 | 732 | -32507.34 | 66478.68 | 0 | 69691.11 | 10460.06 | 10420.56 |
| K80+I | 733 | -30650.85 | 62767.70 | 0 | 65984.51 | 6749.07 | 6713.97 |
| K80+G(4) | 733 | -27382.75 | 56231.50 | 0 | 59448.31 | 212.88 | 177.77 |
| K80+G(4)+I | 734 | -27330.11 | 56128.21 | 0 | 59349.42 | 109.59 | 78.87 |
| HKY | 735 | -32566.75 | 66603.51 | 0 | 69829.10 | 10584.89 | 10558.55 |
| HKY+I | 736 | -30745.06 | 62962.11 | 0 | 66192.09 | 6943.49 | 6921.55 |
| HKY+G(4) | 736 | -27435.08 | 56342.17 | 0 | 59572.15 | 323.54 | 301.60 |
| HKY+G(4)+I | 737 | -27385.23 | 56244.47 | 0 | 59478.84 | 225.85 | 208.29 |
| SYM | 736 | -32451.51 | 66375.01 | 0 | 69604.99 | 10356.39 | 10334.45 |
| SYM+I | 737 | -30586.39 | 62646.77 | 0 | 65881.14 | 6628.15 | 6610.59 |
| SYM+G(4) | 737 | -27331.93 | 56137.86 | 0 | 59372.23 | 119.24 | 101.68 |
| SYM+G(4)+I | 738 | -27281.15 | 56038.31 | 0 | 59277.07 | 19.69 | 6.52 |
| GTR | 739 | -32439.62 | 66357.24 | 0 | 69600.39 | 10338.62 | 10329.84 |
| GTR+I | 740 | -30567.06 | 62614.12 | 0 | 65861.65 | 6595.49 | 6591.11 |
| GTR+G(4) | 740 | -27324.36 | 56128.73 | 0 | 59376.26 | 110.11 | 105.72 |
| GTR+G(4)+I | 741 | -27268.31 | 56018.62 | 1.0000 | 59270.55 | 0 | 0 |

**Table S5.** Detailed model selection results for *GLRaV-3* sequences.

| Model | df | logLik | AIC | AICw | BIC | ΔAIC | ΔBIC |
|---|---|---|---|---|---|---|---|
| JC | 953 | -16570.90 | 35047.80 | 0 | 39633.97 | 2341.86 | 2312.99 |
| JC+I | 954 | -16287.54 | 34483.08 | 0 | 39074.05 | 1777.14 | 1753.08 |
| JC+G(4) | 954 | -16073.71 | 34055.41 | 0 | 38646.39 | 1349.48 | 1325.41 |
| JC+G(4)+I | 955 | -16073.55 | 34057.09 | 0 | 38652.88 | 1351.16 | 1331.91 |
| F81 | 956 | -16571.72 | 35055.45 | 0 | 39656.05 | 2349.51 | 2335.07 |
| F81+I | 957 | -16285.68 | 34485.36 | 0 | 39090.77 | 1779.42 | 1769.80 |
| F81+G(4) | 957 | -16067.42 | 34048.84 | 0 | 38654.25 | 1342.90 | 1333.27 |
| F81+G(4)+I | 958 | -16067.28 | 34050.56 | 0 | 38660.78 | 1344.62 | 1339.81 |
| K80 | 954 | -15925.63 | 33759.27 | 0 | 38350.25 | 1053.33 | 1029.27 |
| K80+I | 955 | -15636.01 | 33182.02 | 0 | 37777.81 | 476.08 | 456.83 |
| K80+G(4) | 955 | -15412.66 | 32735.33 | 0 | 37331.12 | 29.39 | 10.14 |

| | | | | | | | |
|---|---|---|---|---|---|---|---|
| K80+G(4)+I | 956 | -15412.52 | 32737.05 | 0 | 37337.65 | 31.11 | 16.67 |
| HKY | 957 | -15955.06 | 33824.12 | 0 | 38429.53 | 1118.18 | 1108.56 |
| HKY+I | 958 | -15660.13 | 33236.27 | 0 | 37846.49 | 530.33 | 525.52 |
| HKY+G(4) | 958 | -15441.32 | 32798.65 | 0 | 37408.87 | 92.71 | 87.90 |
| HKY+G(4)+I | 959 | -15440.96 | 32799.93 | 0 | 37414.97 | 93.99 | 93.99 |
| SYM | 958 | -15909.69 | 33735.39 | 0 | 38345.61 | 1029.45 | 1024.64 |
| SYM+I | 959 | -15620.48 | 33158.96 | 0 | 37774.00 | 453.02 | 453.02 |
| SYM+G(4) | 959 | -15393.97 | 32705.94 | 0.6940 | 37320.98 | 0 | 0 |
| SYM+G(4)+I | 960 | -15393.89 | 32707.79 | 0.2971 | 37327.64 | 1.85 | 6.66 |
| GTR | 961 | -15924.08 | 33770.15 | 0 | 38394.82 | 1064.22 | 1073.84 |
| GTR+I | 962 | -15628.41 | 33180.82 | 0 | 37810.29 | 474.88 | 489.31 |
| GTR+G(4) | 962 | -15396.98 | 32717.96 | 0 | 37347.43 | 12.02 | 26.45 |
| GTR+G(4)+I | 963 | -15396.86 | 32719.72 | 0 | 37354.01 | 13.78 | 33.03 |

**Table S6.** Best-model summary for nucleotide substitution analyses.

| Virus | BestModel | logLik | AIC | ΔAIC | BIC | ΔBIC | AICw |
|---|---|---|---|---|---|---|---|
| *GFLV* | GTR+G(4)+I | −38188.79 | 78255.58 | 0 | 82500.44 | 0 | 1.0000 |
| *GVA* | GTR+G(4)+I | −27268.31 | 56018.62 | 0 | 59270.55 | 0 | 1.0000 |
| *GLRaV-3* | SYM+G(4) | −15393.97 | 32705.94 | 0 | 37320.98 | 0 | 0.6940 |

**Table S7.** Comparative alignment and bootstrap statistics for three grapevine viruses.

| Virus | Sequences | Length (bp) | Gap % | GC % | Entropy (bits) | BS Mean | BS Median | BS ≥50 % | BS ≥70 % | Model Selected |
|---|---|---|---|---|---|---|---|---|---|---|
| *GFLV* | 466 | 679 | 1.54 | 43.35 | 0.335 | 84.0 | 92.8 | 83.0 % | 65.5 % | GTR + G(4) + I |
| *GVA* | 367 | 595 | 2.63 | 50.33 | 0.381 | 85.0 | 94.4 | 89.9 % | 74.0 % | GTR + G(4) + I |
| *GLRaV-3* | 478 | 909 | 0.44 | 46.27 | 0.167 | 68.3 | 64.3 | 39.7 % | 14.7 % | SYM + G(4) |

Entropy = Mean Shannon H per site; BS = ultrafast bootstrap.

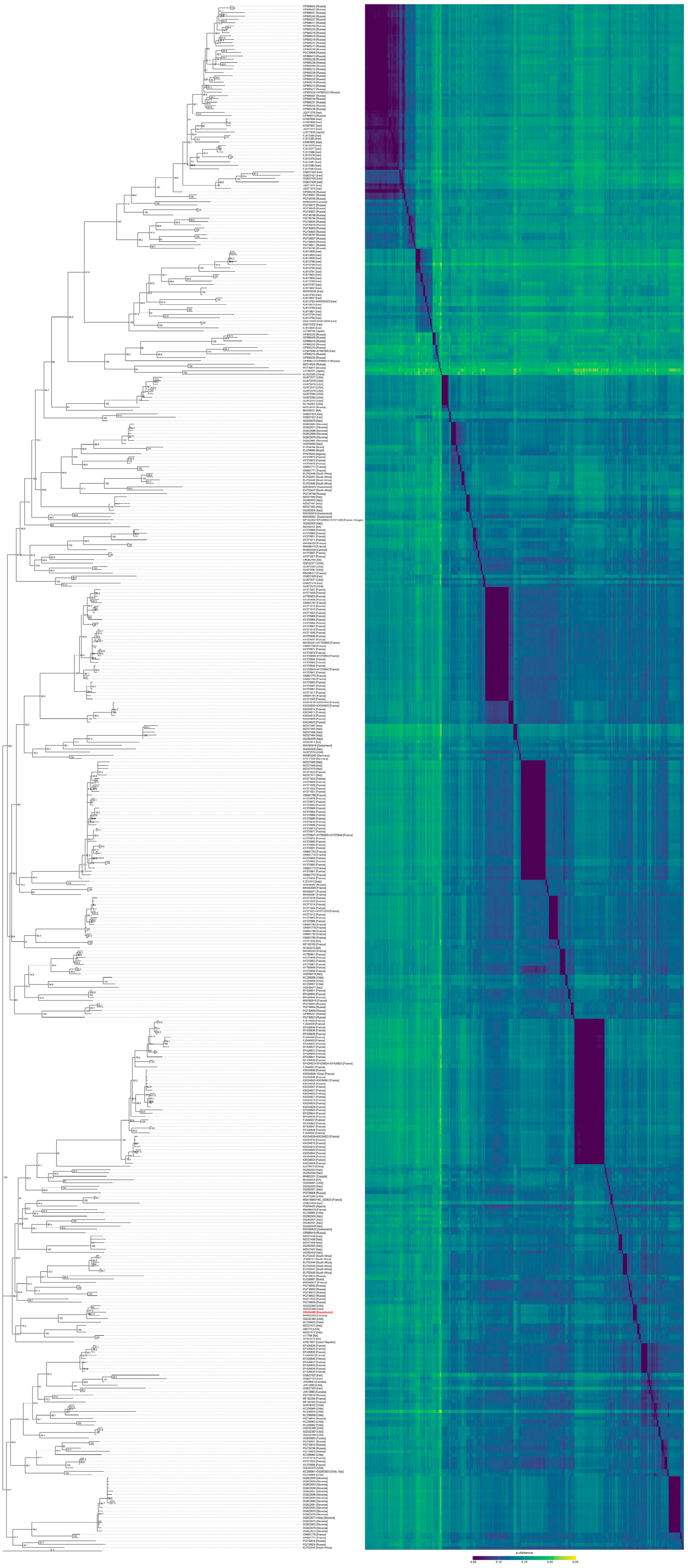

Figure S1. Maximum-likelihood (ML) phylogenetic tree and p-distance heatmap for Grapevine fanleaf virus (GFLV) isolates, including the single Kazakh sequence (OR454495) and 466 international references.

Ivan G. Frolov (frolov.ivg@gmail.com), Karlygash P. Aubakirova, Zhibek N. Bakytzhanova, Akbota Rakhatkyzy, Laura S. Yerbolova, and Nurbol N. Galiakparov

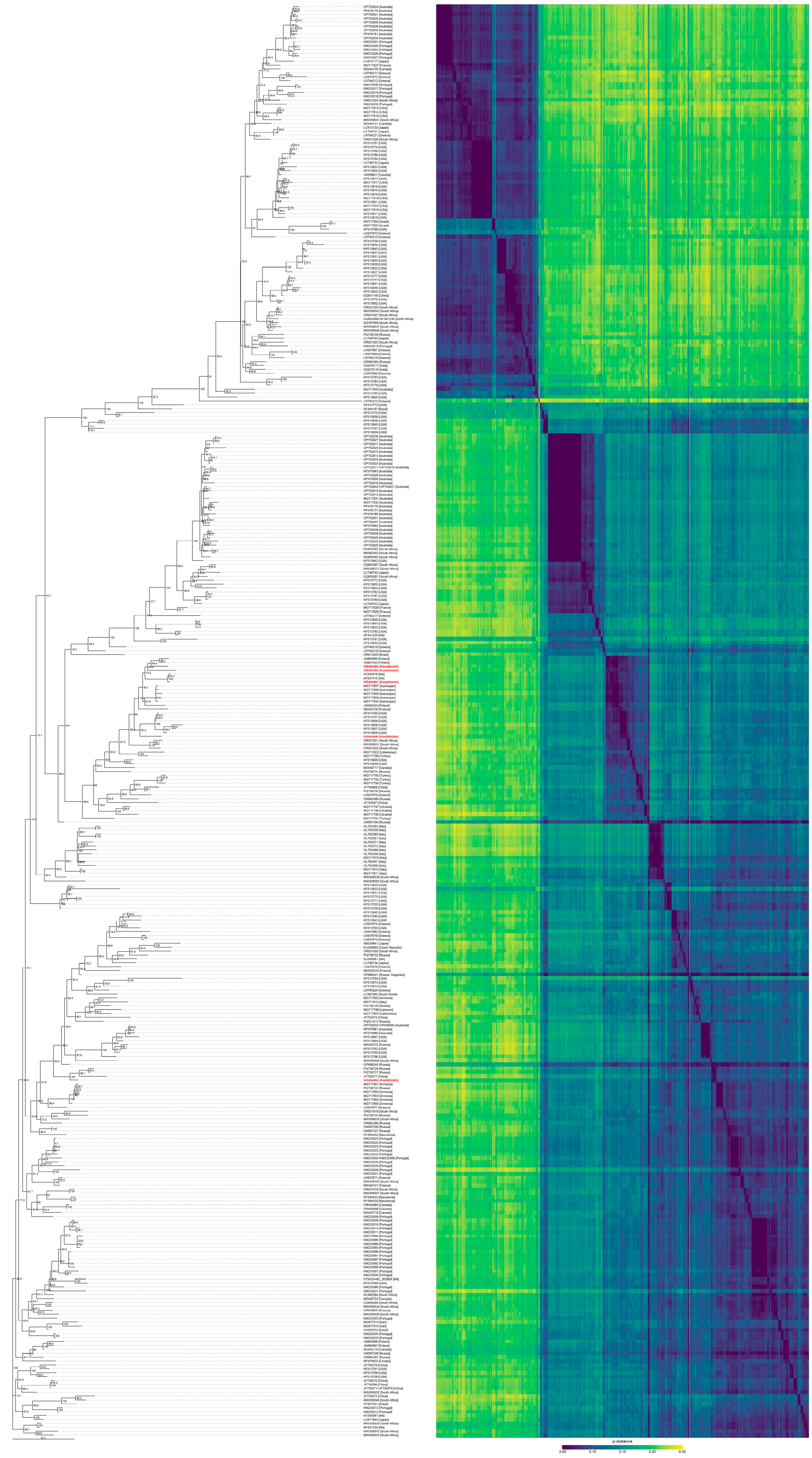

Figure S2. Maximum-likelihood (ML) phylogenetic tree and p-distance heatmap for Grapevine virus A (GVA) isolates, including five Kazakh sequences (OR454490–OR454494) and 367 international references.

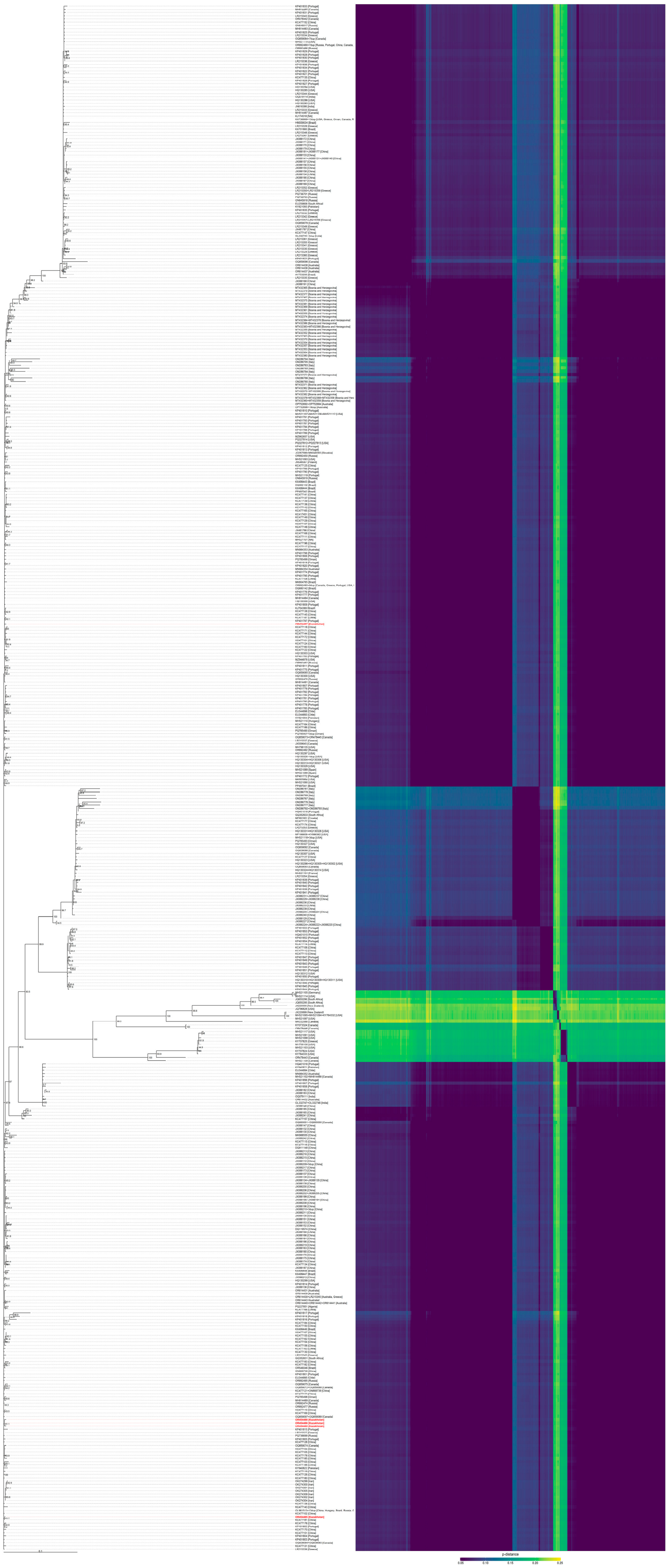


Figure S3. Maximum-likelihood (ML) phylogenetic tree and p-distance heatmap for Grapevine leafroll-associated virus 3 (GLRaV-3) isolates, including five Kazakh sequences (OR454485–OR454489) and 478 international references.
